# COVID-19 in Pregnancy: Implication on Platelets and Blood Indices

**DOI:** 10.1055/s-0041-1733912

**Published:** 2021-09-21

**Authors:** Wassan Nori, Ban Hadi Hameed, Asmaa Rajih Thamir, Amenah Fadhil

**Affiliations:** 1 Department of Obstetrics and Gynecology, College of Medicine, Mustansiriyah University, Baghdad, Iraq; 2 Department of Obstetrics and Gynecology, AL Yarmouk Teaching Hospital, Baghdad, Iraq

**Keywords:** COVID-19, pregnancy, platelet indices, mean platelet volume, platelet distribution width, COVID-19, gravidez, índices de plaquetas, volume médio de plaquetas, largura de distribuição de plaquetas

## Abstract

**Objective** To describe the hematological changes, the platelet indices in particular, in pregnant women with coronavirus disease 2019 (COVID-19) compared to healthy pregnant women.

**Methods** A retrospective case-control study conducted at the Al Yarmouk Teaching Hospital, in Baghdad, Iraq, involving 100 pregnant women, 50 with positive viral DNA for COVID-19 (case group), and 50 with negative results (control group); both groups were subjected to a thorough hematological evaluation.

**Results** Among the main hematological variables analyzed, the platelet indices, namely the mean platelet volume (MPV) and the platelet distribution width (PDW), showed statistically significant differences (MPV: 10.87 ± 66.92 fL for the case group versus 9.84 ± 1.2 fL for the control group; PDW: 14.82 ± 3.18 fL for the case group versus 13.3 ± 2.16 fL for the controls). The criterion value of the receiver operating characteristic (ROC) curve for PDW at a cutoff point of > 11.8 fL showed a weak diagnostic marker, while the MPV at a cutoff value of > 10.17 fL showed a good diagnostic marker.

**Conclusion** The MPV and PDW are significantly affected by the this viral infection, even in asymptomatic confirmed cases, and we recommend that both parameters be included in the diagnostic panel of this infection.

## Resumo

**Objetivo** Descrever as alterações hematológicas, em particular os índices plaquetários em gestantes com doença coronavírus 2019 (COVID-19) em comparação com gestantes saudáveis.

**Métodos** Estudo caso-controle retrospectivo realizado no Hospital Universitário Al Yarmouk, em Bagdá, Iraque envolvendo 100 gestantes, 50 com DNA viral positivo para COVID-19 (grupo caso) e 50 com resultados negativos (grupo controle); ambos os grupos foram submetidos a uma avaliação hematológica completa.

**Resultados** Entre as principais variáveis hematológicas analisadas, os índices plaquetários, nomeadamente o volume plaquetário médio (VPM) e a largura de distribuição plaquetária (PDW), apresentaram diferenças estatisticamente significativas (VPM: 10,87 ± 66,92 fL para o grupo caso versus 9,84 ± 1.2 fL para o o grupo controle; PDW: 14,82 ± 3,18 fL para o grupo caso versus 13,3 ± 2,16 fL para os controles). O valor de critério da curva de característica de operação do receptor (ROC) para PDW em um ponto de corte de> 11,8 fL mostrou um marcador diagnóstico fraco, enquanto o do VPM em um valor de corte de> 10,17 fL mostrou um bom marcador de diagnóstico.

**Conclusão** O MPV e PDW são significativamente afetados por esta infecção viral, mesmo em casos confirmados assintomáticos, e recomendamos que ambos os parâmetros sejam incluídos no painel de diagnóstico desta infecção.

## Introduction

Coronavirus disease 2019 (COVID-19), first described in Wuhan, China, is associated with a rapid inflammatory process and respiratory ailments. The hematological aspects of this infection have also drawn concern, with thrombotic events taking centre stage, showing a distinct concurrent coagulation disorder. 1 The reasons behind this are raised D-dimer, mild thrombocytopenia, and elongated activated partial thromboplastin time. 2 Evidence has shown that platelets are crucial players in promoting inflammation, remodeling, and tissue repair. The role of platelets in viral infection-mediated thrombosis has already been discussed. 3 4 However, the role of platelets in COVID-19 remains ambiguous. Since the platelets do not express the SARS-CoV-2 binding receptor (angiotensin-converting enzyme 2, ACE2), how does the virus interact with the platelets and alter their function? Besides, COVID-19 is characterized by significant inflammation, but reactive thrombocytosis has not been documented. Mild thrombocytopenia is frequently present, and it is a poor prognostic sign. 5 6 Endothelial damage, which is the hallmark of COVID-19 disease, releases the main platelet agonists, sending the platelets into overdrive and altering their morphology and function, which is the justifiable answer for the concerns previously mentioned. 7 Because of the urgent need to recognize the related thrombotic symptoms and the clinical use of impaired coagulation tests to predict the risk and severity of the affection, the present study aims to describe the hematological changes, specially regarding the platelet indices, in pregnant women with confirmed COVID-19, to be included in the catalogue-pathy panel for this disease. The innovative side of the present study is its focus on platelet indices in cases of COVID-19 versus controls to support the underlying pathogenesis for the established high risk of thromboembolism associated with this infection, even in asymptomatic cases.

## Methods

A retrospective case-control study was conducted at Al Yarmouk Teaching Hospital, in Baghdad, Iraq, a tertiary referral hospital with an average of 400 to 600 deliveries per month. The study involved a 100 pregnant women, 50 with confirmed COVID-19 infection (case group), who attended the hospital antenatally, and 50 pregnant women without COVID-19 (control group), who were diagnosed during routine screening and enrolled over the 4 months of the study, from June 1st to October 1st, 2020. We tested both groups for hematological parameters to analyze the blood changes caused by COVID-19.

The medical committee of the College of Medicine of Mustansiriyah University approved the present study (under MOG 114) in July 2020. Informed written consent was obtained from all participants.

**Fig. 1:**
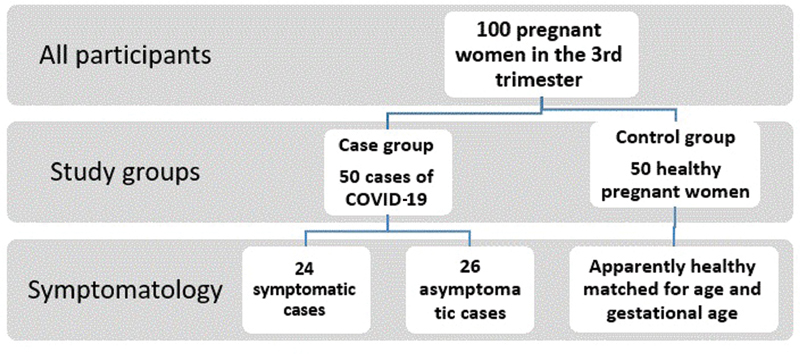
Flowchart of study sample.

All pregnant women who attended the hospital during the study period were preliminarily screened for COVID-19, with a focus on the disease's symptomatology and the patients' history of contact with affected individuals; a detailed clinical examination with all precautions issued by local authorities regarding personal protective equipment was performed. We took throat and nasal swabs of all admitted cases, and tested them for SARS-CoV-2 antigen through the real-time polymerase chain reaction (RT-PCR). The enrolled pregnant women were in the third trimester of pregnancy, and both study groups were matched regarding age, body mass index (BMI). The exclusion criteria were co-morbidities such as preeclampsia and rheumatological diseases, as well as other infections, because they affect hematological variables. A critical case ended with maternal death was excluded to eliminate the statistical bias associated with her extreme laboratory investigation results. Figure 1 shows the flowchart of the study sample.

The sample underwent a complete blood count, including: hemoglobin, total white blood cells (with a differential count of lymphocytes and neutrophils), platelets and their parameters namely the mean platelet volume (MPV) and the platelet distribution width (PDW); the ratios of platelets to lymphocytes and of neutrophils to lymphocytes were assessed and compared between the two groups. We recorded the neonatal birth weight after delivery.

For the statistical analyses, we used the Statistical Package for the Social Sciences (IBM SPSS Statistics, IBM Corp., Armonk, NY, US) software, version 24. The data were expressed as means ± standard deviations/standard errors of the mean. We compare the means of the normally-distributed data by The Student *t* -test. The ROC curve estimated the cutoff point for the significant parameters (MPV and PDW). We set a significant difference when *p* < 0.05.

## Results

The demographics of the sample are described in Table 1 . However, a trend was noticed for the mean gestational age and neonatal birth weight, 36.53 weeks versus 37.5 weeks and 2.65 kg versus 2.86 kg for cases and controls respectively; still, none of the parameters presented a statistically significant difference.

**Table 1:** Demographics of the study groups

Parameter	Case group; *N* = 50 (mean ± SD/SE)	Control group; *N* = 50 (mean ± SD/SE)	*p* -value
Maternal age (years)	27.14 ± 7.75/1.1	27.33 ± 3.79/0.69	0.9
Gestational age (weeks)	36.53 ± 2.71/0.38	37.5 ± 3.2/0.59	0.15
Body mass index (Kg/m ^2^ )	26.61 ± 3.4/0.37	28.45 ± 4.8/0.86	0.98
Neonatal weight (Kg)	2.65 ± 0.61/0.08	2.86 ± 0.56/0.1	0.13

Abbreviations: SD, standard deviation; SE, standard error of the mean.

Table 2 shows the main hematological variables of both groups, and only the platelet indices presented a statistically significant difference: the MPV was of 10.87 ± 66.92 fL in the case group versus 9.84 ± 1.2 fL in the control group, and the PDW was of 14.82 ± 3.18 fL in the case group versus 13.3 ± 2.16 fL in the control group.

**Table 2:** Comparison of hematological parameters between the study groups

Parameter	Case group; *N* = 50 (mean ± SD/SE)	Control group; *N* = 50 (mean ± SD/SE)	*p* -value
Hemoglobin (gm/dl)	11.23 ± 1.54/0.18	11.74 ± 1.3/0.24	0.96
White blood cells (10 ^9^ /L)	11.1 ± 3.78/0.69	11.97 ± 4.93/0.7	0.41
Neutrophil count (10 ^9^ /L)	7.29 ± 3.71/0.53	8.13 ± 3.68/0.67	0.32
Lymphocyte count (10 ^9^ /L)	2.08 ± 0.49/0.9	2.15 ± 0.93/0.13	0.71
Platelet count (10 ^9^ /L)	204.24 ± 66.92/9.56	215.13 ± 54.4/9.9	0.45
Mean platelet volume (fL)	10.87 ± 66.92/9.56	9.84 ± 1.2/0.21	0.002*
Platelet distribution width (fL)	14.82 ± 3.18/0.46	13.3 ± 2.16/0.39	0.024*
Platelet/lymphocyte ratio	110.35 ± 53.21/7.6	107.13 ± 30.79/5.62	0.76
Neutrophil/lymphocyte ratio**	4.14 ± 3/0.42	4.19 ± 2.67/0.48	0.93

Abbreviations: SD, standard deviation; SE, standard error of the mean.

Note: *Statistically significant difference ( *p* < 0.05);

**Fig. 2:**
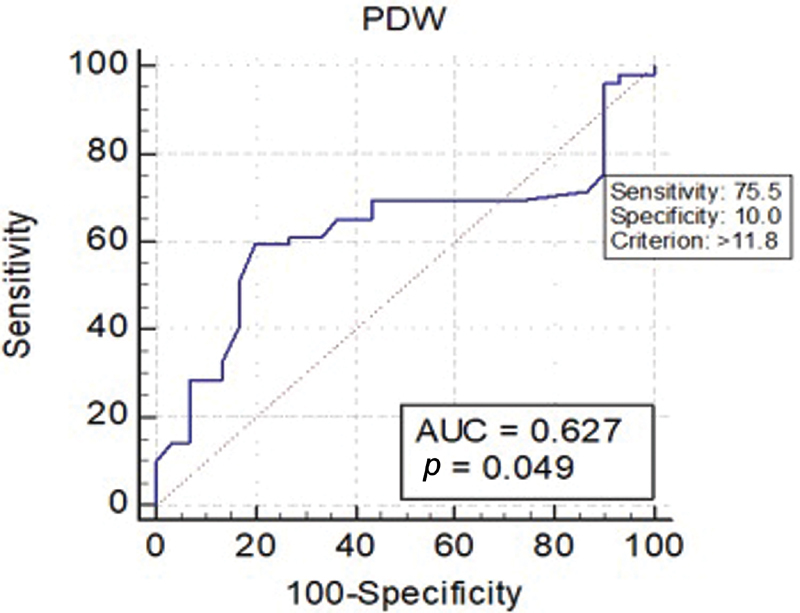
Receiver operating characteristic (ROC) curve for platelet distribution width (PDW).

Figure 2 shows the cutoff value for the PDW (11.8 fL), which was associated with the highest sensitivity (75.5%) and specificity (10%) (95% confidence interval [95%CI]: 0.51–0.73; *p* < 0.05). The area under the curve (AUC) was of 0.627, showing a weak diagnostic marker.

**Fig. 3:**
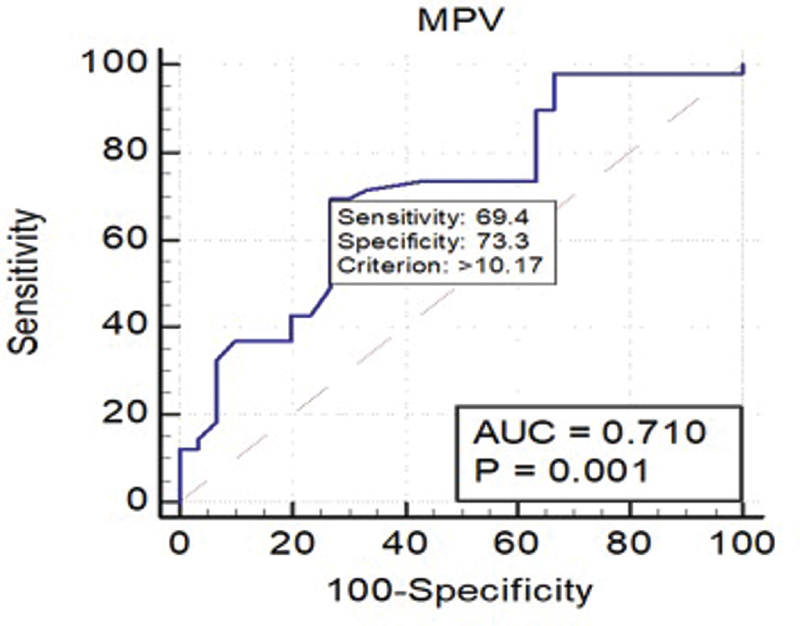
Receiver operating characteristic (ROC) Curve for mean platelet volume (MPV).

Figure 3 shows the cutoff value for the MPV (10.17 fL), which had a sensitivity of 69.4% and a specificity of 73% (95%CI: 0.59–0.80; *p* < 0.05). The AUC was of 0.71, showing an excellent diagnostic marker.

## Discussion

The novel coronavirus has caused serious concerns worldwide, specially regarding its impact on pregnancy and newborn safety. 8 9 The participants' demographics were in line with those described by London et al. 10 regarding maternal age and BMI, as their study included cases of at-term pregnant women who tested positive for COVID-19 and were divided into symptomatic and asymptomatic cases, with no statistical differences between the two groups.

Li et al. 11 analyzed the birth weight of COVID-19 cases versus controls, and showed no significant differences. We can understand this if we recall that all of our participants were mild to moderate cases. Severe cases and cases with pregnancy co-morbidities were excluded from the study by Schwartz et al. 12 The hematological results of the present study were in line with those of the study by Li et al., 11 with no statistically significant difference regarding white blood cells and lymphocytes, and contradicting reports of lymphopenia and leucopenia in hospitalized COVID-19 cases. 13

In the present study, the case group showed a reduced neutrophil count, which is in agreement with the studies by Wang et al. 14 and Manne et al. 15 Derangements in coagulation cascade, such as increased D-dimer, altered fibrinogen level, and platelet level, play a role in thrombotic events in COVID-19. 16 17

Thrombocytopenia secondary to COVID-19 showed a better outcome compared to the outcomes of other more life-threatening viral infections, such as sever cases of influenza, dengue fever, and SARS-CoV-1 infection. 17 18 19 20 Still, it is unclear whether COVID-19 alters the function of the platelets. 20 We verified significant differences between cases and controls regarding platelet parameters in the current study, with the possibility that they play a role in the raised risk of developing thrombosis, which is in line with the findings of the study by Manne et al. 15 The platelet indices in reaction to viral infections were previously assessed in patients with dengue fever in Sudan. 21 Dengue fever showed multiple similarities with COVID-19 regarding seroconversion and the development of immunity. The researchers reported that a low platelet count and MPV, as well as a rising PDW, were good predictors for cases of dengue fevers with a cutoff value less than 9 fl for the MPV and more than 13 fl for PDW; both showed a considerable sensitivity for dengue fever diagnosis. 21

The PDW has been described in the thrombocytosis setting as an indicator of a reactive process, while the MPV mostly unravels the etiology of thrombocytopenia. Identify the risk of developing thrombosis for patients with the ischemic syndrome, and predict the bad outcome after that. Obstetrics had its share of the clinical implications of these parameters', including predicting gestational diabetes, preeclampsia, and adverse neonatal outcomes in high-risk pregnancies. 22

The strength of the present study is that it addresses platelet indices and correlates them with COVID-19 infection as valuable markers to be added to the already-described investigations for the COVID-19 diagnostic panel. Moreover, the present study has a good sample size compared to earlier reports which had shortcomings in sampling and analysis. The limitations are the lack of an analysis of severe and critical cases, and of the impact of altered platelet parameters on maternal and fetal outcomes.

## Conclusion

The MPV and PDW showed significant changes with the COVID-19 infection, even in asymptomatic confirmed cases, and we recommend both parameters to be included in the diagnostic panel of this infection.
